# MicroRNA-221: A Fine Tuner and Potential Biomarker of Chronic Liver Injury

**DOI:** 10.3390/cells9081767

**Published:** 2020-07-23

**Authors:** Jovana Markovic, Amar Deep Sharma, Asha Balakrishnan

**Affiliations:** 1 Department of Gastroenterology, Hepatology and Endocrinology, Hannover Medical School, 30625 Hannover, Germany; markovic.jovana@mh-hannover.de (J.M.); sharma.amar@mh-hannover.de (A.D.S.); 2 Unit miRNA in Liver Regeneration, REBIRTH Research Center for Translational Regenerative Medicine, Hannover Medical School, 30625 Hannover, Germany; 3 Twincore Centre for Experimental and Clinical Infection Research, 30625 Hannover, Germany

**Keywords:** miR-221, liver fibrosis, HCC, NASH, noninvasive biomarker

## Abstract

The last decade has witnessed significant advancements in our understanding of how small noncoding RNAs, such as microRNAs (miRNAs), regulate disease progression. One such miRNA, miR-221, has been shown to play a key role in the progression of liver fibrosis, a common feature of most liver diseases. Many reports have demonstrated the upregulation of miR-221 in liver fibrosis caused by multiple etiologies such as viral infections and nonalcoholic steatohepatitis. Inhibition of miR-221 via different strategies has shown promising results in terms of the suppression of fibrogenic gene signatures in vitro, as well as in vivo, in independent mouse models of liver fibrosis. In addition, miR-221 has also been suggested as a noninvasive serum biomarker for liver fibrosis and cirrhosis. In this review, we discuss the biology of miR-221, its significance and use as a biomarker during progression of liver fibrosis, and finally, potential and robust approaches that can be utilized to suppress liver fibrosis via inhibition of miR-221.

## 1. Introduction

Liver fibrosis and cirrhosis are major contributors to high mortality caused by various liver diseases. The underlying pathogenesis is the activation of quiescent hepatic stellate cells (HSCs) into activated myofibroblasts, which, in turn, secrete excessive extracellular matrix, resulting in liver fibrosis. This is accompanied by hepatocyte dysfunction, apoptosis and necrosis. Due to the presence of diverse autocrine and paracrine fibrogenic regulators, activated myofibroblasts begin to proliferate. To date, multiple molecules have been reported as regulators of HSC activation, such as growth factors including TGFβ, PDGF and FGF, potent cytokines, Toll-like receptors and interleukins. Many of these factors are secreted by hepatocytes and inflammatory cells which reside in or infiltrate into the liver due to liver injury. If it is undetected and remains unresolved, liver fibrosis may develop into cirrhosis and progress to liver cancer such as hepatocellular carcinoma (HCC) or cholangiocarcinoma (CCC). At the molecular level, deregulation of miRNAs is one of the key events in the progression of liver fibrosis and cirrhosis. MiRNAs have been shown to be key regulators of gene expression in liver development and differentiation, as well as in pathophysiological states of the liver. MiRNAs are ~22-nucleotide-long, single-stranded, small noncoding RNAs that are involved in posttranscriptional regulation of gene expression. They are considered regulators of one-third of all human genes, including those involved in fibrogenic pathways. These noncoding RNA molecules target complementary sequences in mRNA transcripts, usually in the 3′ untranslated region (3′ UTR), and prevent protein synthesis, either by inhibiting translation or inducing target degradation. Even though numerous miRNAs have been shown as key regulators of diverse liver diseases and potential biomarkers [1,2], in this review, we focus on miR-221, whose expression is not only upregulated in fibrosis, but also plays a critical role as a driver of liver fibrosis.

In this review, we discuss recent findings concerning the implication of miR-221 in liver fibrosis. First, we summarize the biogenesis and regulation of its expression. We then describe findings that show the role and potential mechanisms of miR-221 during the development of liver fibrosis, and briefly highlight the function of miR-221 in HCC. Finally, we discuss recent evidence of in vivo inhibition of miR-221 and its role as a serum marker for various liver diseases.

## 2. The Biogenesis of miR-221

**Figure 1:**
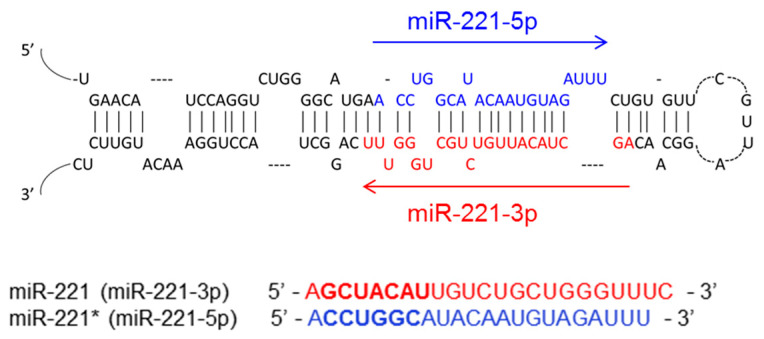
The sequences of miR-221 and miR-221*. Mature sequence of miR-221-5p is in blue, while miR-221-3p is shown in red. The seed sequence of the miRNA is highlighted.

**Figure 2:**
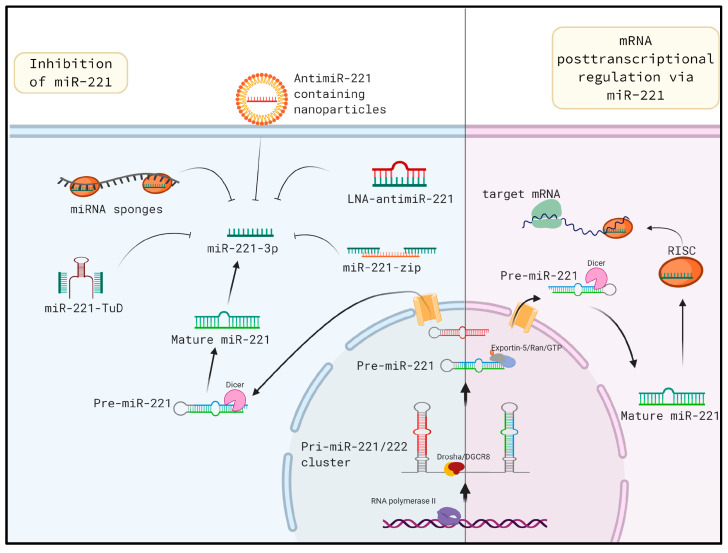
Biogenesis of miR-221 is shown on the right side of the figure. The pri-miR-221/222 cluster is first generated and then processed into pre-miR-221 and pre-miR-222 in the nuclei. Upon translocation of pre-miR-221 from the nuclei to the cytoplasm, Dicer mediates another processing to generate miRNA duplex. One of the strands may be incorporated into RISC complex to regulate gene expression at the posttranscriptional level. On the left side of the schematic, multiple approaches of in vivo miR-221 inhibition are shown. MiR-221 can be inhibited via sponges, antimiR containing nanoparticles, TuDs, miR-zips and LNAs. Each method has been shown to be effective in miR-221 in vivo inhibition. Due to miR-221 inhibition, amelioration of liver fibrosis and attenuation of HCC development was observed.

MiR-221 is encoded and transcribed together with miR-222 as pri-miR-221/222. These two miRNAs are paralogous and share the same seed sequence. The promoter of pri-miR-221/222 is located upstream of miR-222, while three poly(A)-signals are located downstream of miR-221, and the miRNAs are separated by 726 bp. The pri-miR-221/222 is processed in the nucleus by the “microprocessor” complex composed of the enzyme Drosha and RNA binding protein DiGeorge syndrome critical region gene 8 (DGCR8). DGCR8 and Drosha are essential for the nuclear processing of microRNAs; the deficiency of either of them results in the global loss of miRNAs [3,4,5]. Nuclear processing of pri-miR-221/222 leads to the generation of the individual precursors, pre-miR-221 and pre-miR-222. Pre-miR-221 possesses a hairpin and is exported via the exportin5-RanGTP-shuttle system into the cytoplasm where it is further cleaved by the enzyme Dicer into the mature miR-221, whose sequence is shown in Figure 1 [5,6]. The 5′ end of pre-miR-221 gives rise to miR-221-5p, which is also known as miR-221* (star) miRNA. Similarly, the 3′ strand of pri-miR-221 generates miR-221-3p, which is interchangeably used with miR-221. This review focuses on miR-221-3p, which will henceforth be referred to as miR-221. The mature miR-221, upon loading into the RNA-induced silencing complex (RISC), regulates gene expression on a posttranscriptional level by binding to the 3′ UTR of target mRNAs (Figure 2).

## 3. MiR-221 is Upregulated During Liver Fibrosis and Other Chronic Liver Diseases

The indications that miR-221 is upregulated in liver fibrosis and cirrhosis were provided by pioneering studies that sought to analyze the expression of miRNAs in samples isolated from HCC patients who developed cirrhosis [7,8,9]. The elevation in miR-221 expression, among other miRNAs, was subsequently shown in a mouse model of liver fibrosis [10]. However, direct evidence of miR-221 upregulation in livers with hepatitis C virus (HCV), nonalcoholic steatohepatitis (NASH), as well as in in two independent mouse models of liver fibrosis, was provided by Kawada and colleagues [11]. In this study, the authors established a correlation between miR-221/222 expression and fibrosis in patients with HCV who had developed fibrosis. Of note, miR-221 expression was found to be elevated in fibrotic biopsies that showed high levels of collagen expression. This enhanced expression of miR-221 in fibrosis was validated in livers from patients with NASH. In concert with higher miR-221 levels in the HCV group, the authors observed increased miR-221 expression in NASH-associated fibrosis. The expression of miR-221 was examined in HSCs. Indeed, the expression of miR-221 was found to be increased in activated hepatic stellate cells compared to quiescent HSCs. Thus, this study, for the first time, demonstrated enhanced expression of miR-221 in activated HSCs during liver fibrosis.

The excessive accumulation of extracellular matrix during the development of fibrosis causes a decrease in the number of healthy and functional hepatocytes, which eventually leads to liver dysfunction. In addition to molecular changes in HSCs during fibrosis, hepatocytes also undergo dynamic changes at the transcriptomic and epigenetic levels. This includes the upregulation of miR-221 expression in hepatocytes during fibrosis, as it was recently demonstrated by us using purified hepatocytes isolated from fibrotic livers [12]. These results suggest that the upregulation of miR-221 in hepatocytes has functional relevance during fibrosis. In fact, hepatocyte-specific functional knockdown of miR-221 in vivo mitigated fibrosis in two independent mouse models of fibrosis, i.e., induced either by injecting mice with carbon-tetrachloride (CCl_4_) for 8 weeks, or by feeding mice with 3,5-diethoxycarbonyl-1,4-dihydrocollidine (DDC)-containing diet for 4 weeks. Mechanistically, the G-protein alpha inhibiting activity polypeptide 2 (*Gnai2*) posttranscriptional regulation via miR-221 causes a reduced secretion of C-C chemokine ligand 2 (CCL2), which contributes to suppression of liver fibrosis. On one hand, this study highlighted that crosstalk between hepatocytes and HSCs indeed directs fibrogenesis in an injured liver. On the other hand, these results suggested that miR-221 modulation in hepatocytes, the parenchymal cells making up over 65% of liver mass, possesses the potential for mitigation of fibrosis. Thus, both HSCs and hepatocytes show enhanced levels of miR-221 expression in response to fibrosis, and inhibition of miR-221 in both cell types can suppress fibrosis.

Subsequent studies have found upregulation of miR-221 in HCC, suggesting a correlation between high miR-221 expression and different cellular oncogenic pathways. One such pathway is Nuclear Factor kappa-light-chain-enhancer of activated B cells (NF-kB), the activation of which may be promoted by miR-221 in HCC [13]. Furthermore, in this study, the authors demonstrate that miR-221 inhibition results in reduction of metastasis, increased apoptosis, reduced tumor growth, and inactivation of the NF-kB pathway, while the re-introduction of miR-221 leads to the gain of tumor properties. Pineau et al. were able to show increased expression of miR-221 along with 11 other miRNAs in HCC tissues. Moreover, following treatment with anti-miR-221, decreased colony formation was observed in a HCC cell line, while transplantation of *p53^-/-^myc* liver progenitors with overexpressed miR-221 was shown to enhance cell proliferation and tumor growth. Further confirmation of high miR-221 levels in HCC tissues came from Li et al., who identified miR-221 as a regulator of epithelial-mesenchymal transition (EMT) in cancer cells in vitro [14]. An inhibitor of the JAK/STAT pathway, AdipoR1, was identified as a novel target, and the authors correlated tumor size, vascularity and the number of tumors with the level of miR-221. The implication of miR-221 through JAK/STAT modulation in HCC has also been shown by Huang et al. [15]. The authors confirmed previous findings where increased miR-221 levels in advanced stages of HCC influenced the invasion and migration of tumors, and additionally discovered *SOCS3*, an inhibitor of the JAK/STAT pathway, as a target of miR-221. They showed that *SOCS3* expression is downregulated in HCC patients, as well as in cells transfected with miR-221, and that in vitro miR-221 inhibition or *SOCS3* overexpression inhibits tumor growth.

## 4. Regulation of miRNA-221

Two transcription factors, NF-kB and c-Jun, have been shown to regulate the transcription of miR-221 by the Ciafre group [16]. The authors first demonstrated that ectopic modulation of NF-kB leads to altered miR-221 expression. Using bioinformatic prediction tools, two separate distal regions that NF-kB binds with were identified upstream of the miR-221 promoter region. Using electrophoretic mobility shift assays and chromatin immunoprecipitation experiments, the authors provided strong evidence of binding of NF-kB to the promoter of miR-221. Furthermore, the c-Jun binding site was found in the most distal enhancer region of miR-221. Subsequently, using siRNA experiments, the authors provided functional data for miR-221 regulation by both transcription factors. Importantly, both transcription factors have been shown to play critical roles in the development of liver fibrosis and HCC [17,18,19,20]. NF-kB inhibition leads to apoptosis in HSCs and reduction of extracellular matrix formation. It has also been suggested that c-Jun promotes liver fibrosis [21,22]. Therefore, upon liver damage that leads to fibrosis, NF-kB and c-Jun act as initiators of the molecular cascades that regulate apoptosis and inflammation and concomitantly increase miR-221 expression. Additionally, MET has been shown to regulate miR-221/222 expression by Garofalo et al. [23]. The authors showed that AP-1, a transcription factor from the Jun family which is involved in the c-Met pathway, binds and transcriptionally activates the miR-221/222 promoter. Subsequently, MET knockdown was demonstrated to downregulate miR-221 expression. Since miR-221 overexpression and MET pathway activation have been found in many cancers, the authors demonstrated that via both MET- and miR-221- knockdown, there is an increase in levels of tumor suppressor proteins, such as phosphatase and tensin homolog (PTEN).

## 5. Potential Mechanism of Fibrosis Regulation by miR-221

The function of miR-221 is cell-type dependent; it has been suggested that it acts either to promote or inhibit proliferation [24]. In the case of fibrosis, in addition to the posttranscriptional target, *Gnai2* [12], miR-221 has been shown to regulate multiple other targets, and therefore, affect fibrogenesis directly or via modulation of HSC proliferation. Cyclin-dependent kinase inhibitors such as *CDKN1C* and *CDKN1B* are posttranscriptional targets of miR-221 that were discovered in liver cells [8]. The downregulation of *CDKN1B* enhances proliferation of HSCs [25], and the gain of *CDKN1C* function in HSCs inhibits their proliferation [26]. Thus, CDKN1C and CDKN1B are able to control the proliferation of HSCs, and hence, affect fibrogenesis. In addition, p53 Up-Regulated Modulator of Apoptosis (*Puma*) and Aryl Hydrocarbon Receptor Nuclear Translocator (*Arnt)*, two targets of miR-221, have been shown to regulate hepatocyte apoptosis [3] and proliferation [27], respectively. Similar to miR-221-mediated *Gnai2* modulation in hepatocytes, it is plausible that *Puma* and *Arnt* may affect fibrogenesis via inhibiting apoptosis of hepatocytes and enhancing proliferation. However, it remains to be examined if change in the expression of *Puma* and *Arnt* in hepatocytes influences fibrosis via HSCs or other cell types in the liver.

Suppressor of Cytokine Signaling (*Socs1*), another target of miR-221 [28], has been shown to regulate fibrogenesis by limiting damage to hepatocytes and the inflammatory response of macrophages [29]. Hepatocyte-specific depletion of *Socs1* in mouse liver caused a higher degree of fibrosis upon CCl_4_ administration, as characterized by increased collagen and hydroxyproline contents and the accumulation of myofibroblasts with high levels of smooth muscle actin (α-SMA), an indicator of HSC activation. Similarly, the macrophage-specific depletion of *Socs1* led to increased fibrogenesis. Both the lack of SOCS1 in hepatocytes and in macrophages resulted in increased infiltration of immune cells and high levels of CCL2, a known regulator of fibrosis [30]. It is noteworthy that two targets of miR-221, *Gnai2* and *Socs1*, converge to support similar findings of upregulation of CCL2 as one of the driving forces of fibrosis. These findings further highlight the relevance of miR-221 and CCL2 during fibrogenesis.

miR-221 has been shown to regulate the expression of E-cadherin at the posttranscriptional level [31]. The inverse correlation of elevation of miR-221 and downregulation of E-cadherin during chronic liver diseases is frequently observed. The reduction in E-cadherin expression is one of the features of EMT. Early embryogenesis includes Type 1 EMT, while Type 2 EMT is associated with the fibrogenesis process, and Type 3 EMT is often observed during metastasis of neoplastic cells. It has also been suggested that Type 2 EMT may play a key role in the cycles of wound healing and inflammation during liver fibrosis [32,33]. Twist homolog 2 (TWIST2) [34] and Slug [31], both transcription factors known to drive EMT, have also been shown to regulate miR-221 expression. In fact, recent evidence demonstrated that miR-221 indeed regulates EMT in end-stage liver diseases [14,35]. Despite earlier reports that highlighted the relevance of EMT in liver fibrosis, subsequent reports in lineage tracing transgenic mouse models suggested that EMT only plays a limited role in fibrosis [36,37]. Therefore, it is critical to examine whether miR-221 indeed induces EMT, especially when modulated in hepatocytes, and to what extent the inhibition of EMT contributes to the attenuation of liver fibrosis by miR-221 knockdown.

Phosphatase and tensin homolog (*PTEN*), an inhibitor of proliferation, has been shown to be a posttranscriptional target of miR-221 [23,35]. The loss of *Pten* in the liver results in progressive liver fibrosis with higher expression of fibrogenic markers such as TIMP1, p75NTR, Collagen and SMA [38]. At the cellular level, isolated HSCs from *Pten* null livers contain lower numbers of vitamin A positive cells and increased expression of *Acta2*, indicating increased activation of *Pten^-/-^* HSCs compared to HSCs isolated from the wild-type livers.

TIMP3 has been shown to be an important regulator of liver fibrosis, inflammation and steatosis through the modulation of TNF-α-converting enzyme (TACE), and is therefore considered to play a key role in NASH and NAFLD development. The regulation of *Timp3* mRNA expression by miR-221/222 has been shown by Jiang et al. [39]. The authors demonstrated that upon hepatocyte-specific *miR-221/222* knockout, downregulation of expression of fibrogenic markers such as *Col1a1*, *Col1a2*, *Col3a1* and *Acta2*, hepatogenic TG content and lipid droplet deposition are seen in both the methionine and choline deficient diet (MCDD)-induced NASH model and CCL_4_-induced liver fibrosis. Additionally, the authors detected increased expression of the miR-221/222 cluster in a NASH model, while *Timp3* expression was downregulated. The inverse correlation between the expression of miR-221/222 and TIMP3 was further confirmed by Garofalo et al. [23]. The authors confirmed *TIMP3* as a miR-221/222 target in HCC-derived cancer cells and showed that as a consequence of miR-221-mediated *TIMP3* degradation, these cells were resistant to treatment with TNF-related, apoptosis-inducing ligand (TRAIL).

DDIT4, a regulator of the mammalian target of rapamycin (mTOR) kinase, has been shown as an important protein in tumor development due to its tumor suppressing properties. *DDIT4* expression is regulated by miR-221 in vitro and in vivo [9]. The authors showed that miR-221 is overexpressed in both HCC and cirrhotic human livers, and enhances tumor growth through *DDIT4* targeting and deregulation of the PI3K-PTEN-AKT-mTOR pathway. Interestingly, both *DDIT4* and *PTEN* are shown as miR-221 targets and are separated by only 12Mb.

miR-221 has been found to increase β-catenin signaling by targeting Wnt target gene axis inhibition protein 2 (*AXIN2*), a protein involved in β-catenin degradation [40]. Dong et al. found increased expression of miR-221 and miR-15b in liver cancer tissues, and showed *AXIN2* as a common target of both miRNAs in HCC cell lines. There was a decrease in proliferation and invasion of HCC cells upon miR-221/miR-15b knockdown or *AXIN2* overexpression. Of note, Wnt signaling in hepatocytes is known to affect the expression of fibrogenic genes upon co-culture with HSCs [41].

Another important target of miR-221 is Bcl-2 modifying factor (*BMF*), an apoptotic activator whose expression level is in direct correlation with the caspase pathway [7]. Gramantieri et al. showed that *BMF* mRNA is a target of miR-221 in vitro, wherein their in vivo HCC expression is inversely correlated. Furthermore, the authors demonstrated higher expression of miR-221 and a corresponding decrease in expression of *BMF* in multifocal HCC compared to unifocal HCC. Accordingly, the role of miR-221 in HCC development was established, not only as a cell cycle modulator, but also as an anti-apoptotic regulator. Besides HCC, *BMF* has also been shown to be a miR-221 target in ovarian cancer cells [42].

## 6. In vivo miR-221 Inhibition: A Potential Approach for Mitigating Liver Fibrosis

### 6.1. Tough Decoys

The downregulation of miR-221 presents a plausible approach for the amelioration of liver fibrosis. Therefore, multiple approaches for miR-221 knockdown have been attempted. One such approach is to use tough decoys (TuD). TuD constructs contain bulge hairpin and two miRNA target sites which imperfectly pair with the target miRNA (Figure 2) [43]. TuDs have been used to efficiently downregulate multiple miRNAs including miR-221 [44]. Our laboratory adapted this method to downregulate miR-221 expression in mouse models of liver fibrosis [12]. Specifically, miR-221-TuD was cloned in an AAV expression plasmid under a hepatocyte-specific promoter, and high titer AAV vectors were prepared. In vivo knockdown of miR-221 upon AAV administration led to downregulation of fibrogenic genes such as *Col1a1, Acta2* and *Desmin,* and reduced cell infiltration in the fibrotic area. Thus, miR-221-TuD delivered via AAV vector into a damaged liver is capable of attenuating fibrosis. MiR-221-TuD expressed from an AAV vector was tested in an additional study by Ma et al. [45]. The authors compared the miR-221-sponge, miR-221-TuD and miR-221-zip for their potential to inhibit miR-221 in vitro. After finding that miR-221-TuD and miR-221-zip were more potent, the authors then administered the inhibitors in vivo via intratumoral AAV injections into xenograft tumors which were generated from HepG2 cells in BALB/c nude mice. Upon miR-221 inhibition, a significant effect was observed, with mice having smaller tumor burdens and showing increased TRAIL-induced apoptosis.

### 6.2. Locked Nucleic Acids

Besides TuD, locked nucleic acids (LNA) can be used for a miRNA knockdown. LNAs are nucleic acids in which some of the ribose molecules within the nucleotides contain a methylene bridge that connects 2′ oxygen and 4′ carbon. Due to the methylene bridge, the ribose is locked in the C3’-endo or C2’-endo conformation [46]. LNAs can be designed to bind to DNA and RNA molecules. LNA-antimiRs are antisense RNAs in which usually every third nucleotide has a modified ribose moiety, and which possess a much higher hybridization affinity and stability in miRNA-LNA-antimiR duplexes than traditional probes [47]. In the miRNA-LNA-antimir duplex, miRNA function is antagonized [48]. The best-known LNA-antimiR is miravirsen (Santaris Pharma), an LNA antisense to miR-122 currently in clinical trials for the treatment of patients with hepatitis C infection [49]. MiR-122, the most abundant miRNA in the liver, has been shown to bind to the 5′ UTR of the hepatitis C virus RNA, which is conserved across all six genotypes of the virus, facilitating its replication [50]. Multiple studies have successfully used LNAs to ameliorate fibrosis in organs, including the kidney, heart and liver [51,52,53,54].

Since miR-221 is also a key regulator of NASH, Jiang et al. showed in vivo potential of LNA-antimiR-221 in reducing hepatic steatosis in the MCDD mouse model of NASH [39]. Upon in vivo administration of LNA-antimiR-221, hepatic TG content, inflammation, steatosis and hepatocyte ballooning were decreased. In concert with other studies, Jiang et al. also reported amelioration of fibrosis.

### 6.3. MiRNA Sponges

An additional approach for miRNA inhibition in vivo and in vitro is with miRNA sponges. MiRNA sponges contain multiple sequences that are complementary to miRNA seed sequences, resulting in miRNAs binding to the sponge instead for the target mRNA, which leads to miRNA sequestration [55]. When compared to other miRNA inhibitors, such as TuD or LNA, which require nucleotide complementarity beyond the seed sequence, sponges offer the advantage of inhibiting multiple miRNAs that share a common seed sequence. Also, miRNA sponge expressing transgenic animal models can be developed, providing lifelong inhibition of miRNAs. When it comes to clinical applications, these features are not desirable, since it is preferable to reversibly inhibit only one miRNA. Therefore, in clinical settings, oligonucleotides have been more widely used.

Moshiri et al. designed a miR-221 sponge that contains four miR-221 binding sites inserted in both the adenoviral and AAV vectors [56]. They selected a conditionally replicative oncolytic adenovirus whose replication was under the control of miR-199a, a miRNA moderately expressed in healthy livers but downregulated in HCC. They also developed a rAAV vector containing a miR-221 sponge. The inhibition potential of the miR-221 sponge was tested in vitro using HCC cell lines and assessed by the level of CDKN1B (p27), which is a known target of miR-221. The authors showed increased expression of *p27* in the presence of the sponge and increased apoptosis. Even though the authors did not test it in vivo, they assumed that both approaches could be used. Naturally occurring sponges for miRNAs are long noncoding RNAs. One such RNA is *CASC2*, which was shown by Jin et al. to act as a sponge for miR-221 in HCC cell lines [57]. The authors predicted the binding of *CASC2* with miR-221 bioinformatically and experimentally. They also demonstrated caspase-3 as a miR-221 target in HCC cells, and an inverse expression of miR-221 and *CASC2*, which was known as an inhibitor of cancer cell proliferation. Furthermore, it was shown that *CASC2* modulated TRAIL resistance through miR-24/miR-221 and increased levels of caspase-3. Lately, nonviral systems such as nanoparticles are emerging as promising therapeutic approaches because of their low toxicity. Such a system was employed in a study by Li et al., who used nanoparticles with miR-221 mimic/inhibitor, both in vivo and in vitro, in order to track HCC development. Nanoparticle-based miR-221 inhibitors decrease cell proliferation, colony formation ability and tumor development, and result in lower serum levels of miR-221, thus representing a promising approach for HCC therapy [58]. Additionally, Cai et al. demonstrated that a synergistic effect is achieved with the usage of a combination treatment regimen of nanoparticles containing anti-miR-221 and chemotherapeutic drugs, such as sorafenib [59]. Specifically, the authors tested the combination in HCC cell lines where cell proliferation arrest was achieved. Even though their data provided initial evidence that the antimiR-221 encapsulated nanoparticles should be able to treat solid tumors, further experiments in vivo are needed for confirmation.

### 6.4. MiR-221 as A Serum Biomarker

**Figure 3:**
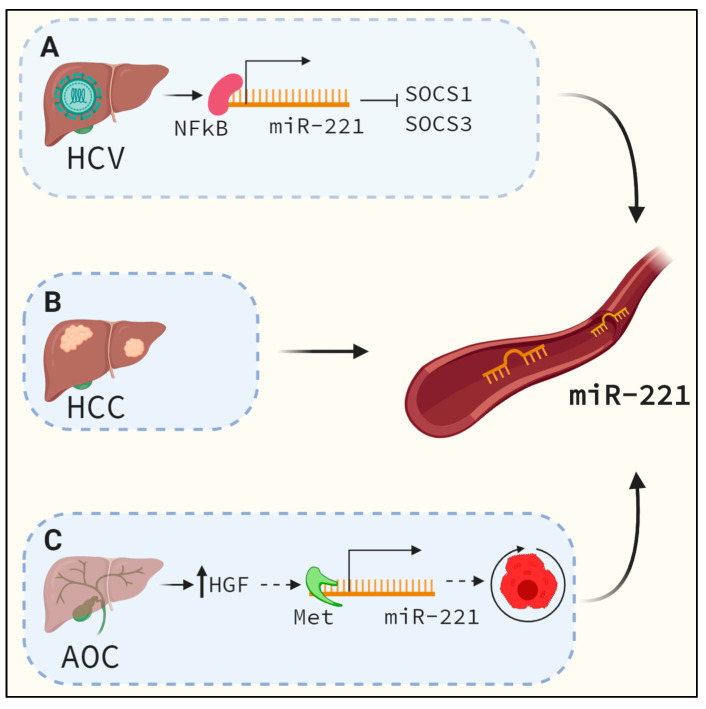
The main etiologies of liver fibrosis are chronic viral hepatitis infection, NASH and cholestatic diseases. If left untreated, extensive liver fibrosis often leads to the development of HCC. At present, the most commonly used method to diagnose liver disease is biopsy, which is invasive and often time-consuming. More importantly, the biopsy is often performed at later stages, by which time the only effective treatment option is transplantation. MiR-221 has been shown as a miRNA that can serve as a serum biomarker for diagnosis, to distinguish different diseases and as a marker of recovery. (**A**) HCV is one of the most prevalent causes of liver fibrosis. Multiple studies have shown miR-221 upregulation in the sera of patients with HCV. Furthermore, in HCV, miR-221 expression is under the control of NF-Kb, and it targets *SOCS1* and *SOCS3*. (**B**) HCC is the fourth leading cause of cancer-related deaths worldwide. High levels of miR-221 have been detected in HCC tissues and in the sera of patients with HCC. Based on the serum levels, miR-221 can be used together with AFP as a biomarker of HCC development, severity and outcome. Patients with high miR-221 had more advanced HCC and lower survival rates than those with low miR-221 serum levels. (**C**) In contrast, in AOC, the upregulation of miR-221 in patient’s sera is considered a sign of recovery. In AOC, hepatocyte proliferation and liver regeneration occur due to high HGF signaling. HGF is the key ligand for the receptor tyrosine kinase, c-Met, which, in turn, enhances miR-221 transcription. High levels of miR-221 in the liver repress *SOCS1* levels. Since SOCS1 is an inhibitor of HGF signaling, its repression by miR-221 leads to enhanced HGF signaling and active liver regeneration and recovery.

To date, liver biopsy remains the gold-standard method for diagnosing liver fibrosis and end-stage liver diseases. Among noninvasive approaches, analyses of biomarkers such as circulating miRNAs hold great potential for diagnosis. Higher stability of miRNAs in the serum and plasma, as well as the fact that a change in their expression in circulation often precedes injury, make them promising candidates as biomarkers [60,61]. One of the most common causes of liver fibrosis is chronic liver damage due to HCV infection. In the serum of HCV infected patients, increased levels of miR-221 were detected by Ding et al. (Figure 3) [62]. The authors showed a correlation between increased levels of miR-221, ALT and AST. The expression of miR-221 was shown to be controlled by NF-kB, as had been previously demonstrated in other studies. Findings from this study were confirmed by Xu et al. [63]. Additionally, the authors identified *SOCS1* and *SOCS2* as miR-221 targets (Figure 3). HCV infection can often lead to the development of liver cirrhosis, and eventually, to liver cancer, which is the fourth most common cause of cancer related deaths worldwide. High expression of miR-221 has been demonstrated in HCC tissues and in serum. Sohn et al. showed high levels of miR-221 among other miRNAs in serum exosomes and as a circulating miRNA in patients with HCC and chronic HBV infection [64]. The authors showed that the level of miR-221 in serum exosomes could be used to distinguish between HCC and chronic hepatitis B (CHB). While analyzing circulating levels of miR-221 in the serum, the authors did not find a difference between the HCC and CHB groups. It is important to note that the authors did not correlate serum miR-221 levels with tissue expression. Furthermore, Li et al. found significantly increased levels of miR-221 in HCC serum samples [65], and showed a correlation between the level of miR-221 and HCC severity and disease outcome. The patients with high miR-221 serum levels had more advanced cirrhosis, tumor size and stage. Additionally, the survival rate was significantly lower than in patients with low miR-221 serum levels. The authors, however, implied that the combination of miR-221 and AFP serum levels provides a better diagnostic prediction than the use of each of these markers separately. Other studies have also confirmed these findings [66,67]. In contrast, in acute obstructive cholangitis (AOC), increased levels of miR-221 in patients’ sera were shown to be a positive sign of recovery [68]. In AOC, a biliary obstruction occurs, followed by an infection that leads to inflammation. There is also a regenerative response that is triggered in hepatocytes through enhanced HGF signaling. HGF is the primary ligand for the receptor tyrosine kinase and oncogene, c-Met [69], which, in turn, enhances miR-221 transcription (Figure 3). In AOC, high miR-221 is indicative of recovery. The expression of miR-221 in hepatocytes during AOC is regulated by the SOCS1–Met axis. Downregulation of SOCS1 increases the expression of miR-221. It has been shown that SOCS1 inhibits HGF signaling and attenuates HGF mediated phosphorylation of c-Met in hepatocytes. Due to the chronic inflammation which occurs in AOC, SOCS1 is upregulated. However, during regression of AOC, the authors showed that SOCS1 levels reverted to normal levels, while Met and phospho-Met levels were increased. Phosphorylated c-Met enhances miR-221 transcription, whose overexpression has been shown to accelerate hepatocyte proliferation during liver regeneration (Figure 3) [27,70,71,72]. Thus, increased miR-221 expression indicates active liver regeneration and recovery.

High levels of miR-221 in serum in HCC/HCV are correlated with advanced-stage diseases, but these same high levels in AOC are indicators of recovery. Even though the increase in miR-221 levels is triggered by different mechanisms—in HCC/HCV, its expression is mostly regulated by NFkB, while in AOC, it is accomplished via HGF—in both instances, the role of miR-221 is to accelerate cell proliferation. Therefore, the dichotomous functional relevance of elevated miR-221 levels allows for its use as a prognostic biomarker in HCC/HCV disease progression or AOC recovery monitoring.

## 7. Unanswered Questions

Recent research has unequivocally established miR-221 as a key regulator of liver fibrosis. However, the following questions, especially pertaining to the prerequisites to the use of miR-221 inhibitors to attenuate liver fibrosis in a clinical setting, remain:

The expression of miR-221 in nonparenchymal cells such as Kupffer cells has been shown to be higher during fibrosis than in the normal healthy liver. Whether increased levels of miR-221 in Kupffer cells contribute to progression of liver fibrosis remains to be investigated.

The knockdown of miR-221 in HSCs and hepatocytes mitigates fibrogenic gene expression. However, it remains to be seen whether combined suppression of miR-221 in hepatocytes, HSCs and other nonparenchymal cells reduces fibrosis to a greater extent.

To date, most studies have focused on the identification of miR-221 as a regulator of liver fibrosis. Given that fibrosis, if left unchecked, may develop into cirrhosis, the reversal of cirrhosis upon miR-221 inhibition must be demonstrated in order to translate the present findings regarding miR-221 into therapeutic pathways.

Likewise, the relevance of miR-221 in mouse models that develop HCC after fibrosis and cirrhosis remains to be examined. Hence, it would be extremely important to study whether the inhibition of miR-221 in those models in fibrotic or cirrhotic stages prevents progression to HCC.

Multiple studies have suggested a potential application of miR-221 as a biomarker of liver fibrosis. However, the number of patients in cohorts remains low; therefore, multicenter studies are required before miR-221 can be used as a reliable serum biomarker of liver fibrosis and cirrhosis.

## 8. Conclusions

Over the last few years, tremendous progress has been made towards elucidating the function of miRNAs during liver fibrosis. MiR-221 has emerged as a key regulator that is upregulated during liver fibrosis caused by various etiologies. Studies in mouse models of fibrosis have provided ample evidence that intrahepatic suppression of miR-221 holds significant potential for the mitigation of liver fibrosis. However, further research is required to specifically address the effects of miR-221 inhibition in cirrhosis, its use as a serum biomarker, and the safety, including potential side effects, of miR-221 modulation in damaged livers.
